# Supplementary material for: Phase transformation strengthening of high-temperature superalloys
Source: Nat Commun. 2016 Nov 22;7:13434. doi: 10.1038/ncomms13434 (PMC5121413; doi:10.1038/ncomms13434)
Supplement: Supplementary Information — Supplementary Figures 1-7 and Supplementary Tables 1-5 and Supplementary Notes 1-4 and Supplementary References [file ncomms13434-s1.pdf]

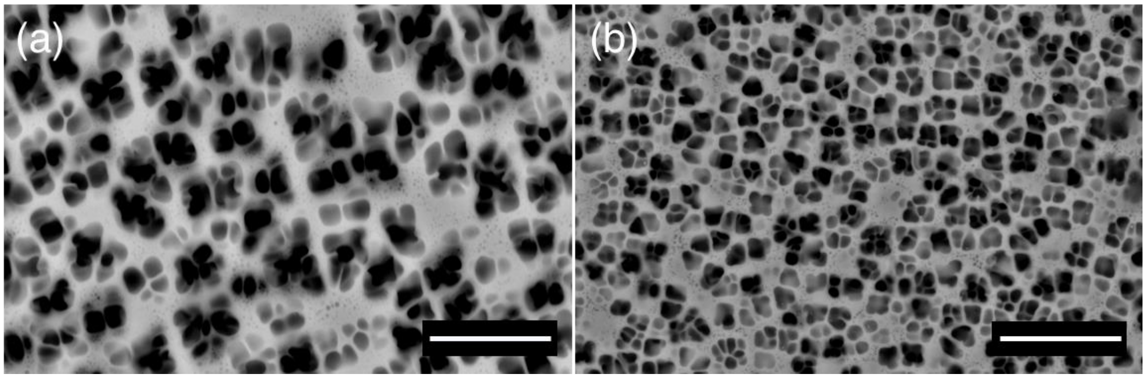

Supplementary Figure 1: Microstructural Characterization of ME3 and ME501. SEM backscatter image of the (a) ME3 and (b) ME501 microstructures. All scale bars, 500nm.

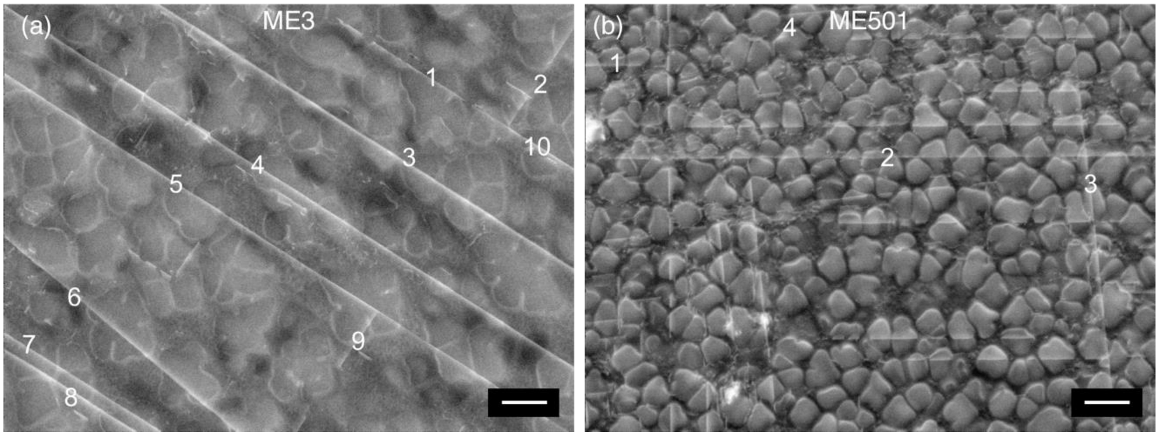

Supplementary Figure 2: Electron channeling contrast images of nanotwins in ME3 and ME501. ECCI image of (a) ME3 showing 10 different twins and (b) ME501 showing 4 different twins. All scale bars, 500nm.

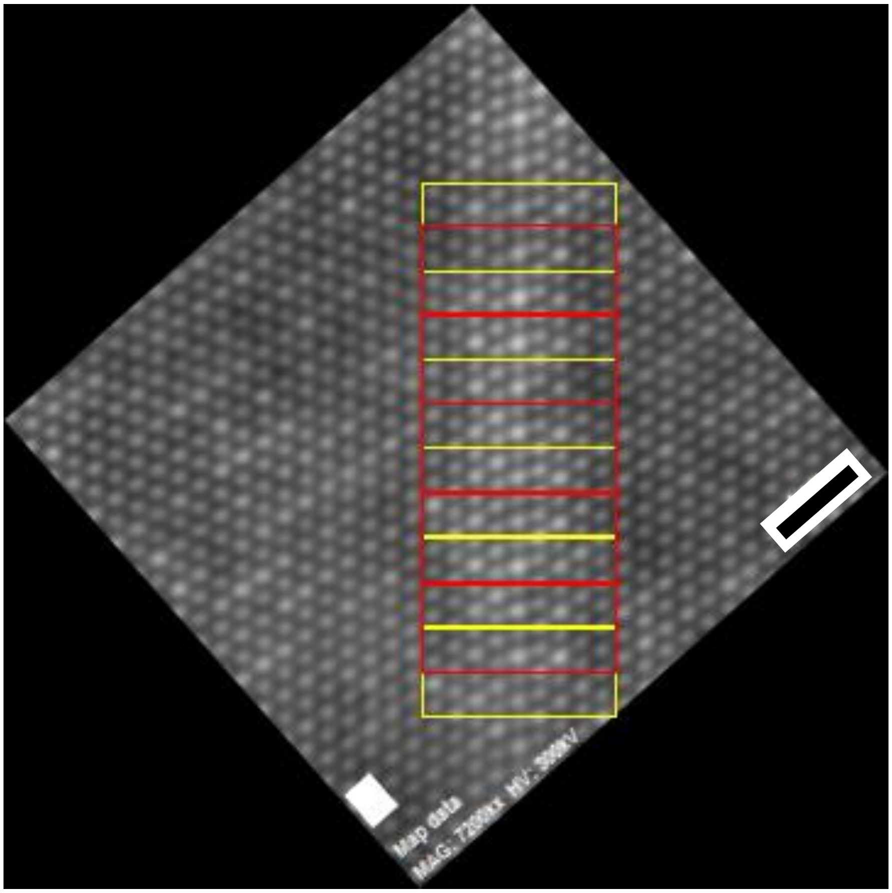

Supplementary Figure 3: Repeating unit cells along a SESF. Rotated EDX spectrum image, showing the HAADF layer with 6 repeating units along the SESF (yellow boxes) and 5 additional repeating units (red boxes) taken midway between the first 6. The scale bar is 1 nm.

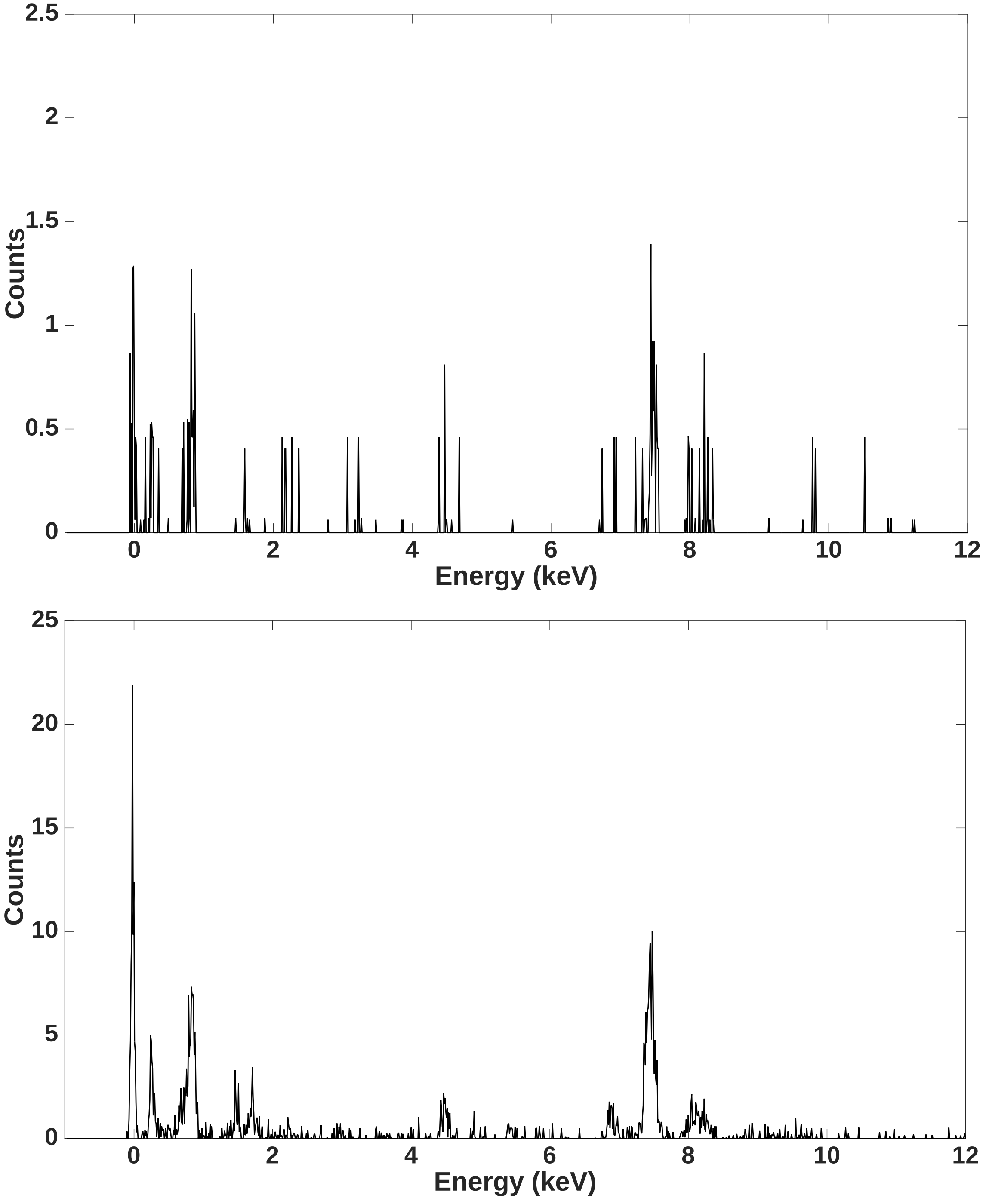

Supplementary Figure 4: EDX Spectra of raw and summed data. Representative EDX spectra from raw data (top) and summed data (bottom) showing a significant improvement in both counts per peak and signal-to-noise ratio

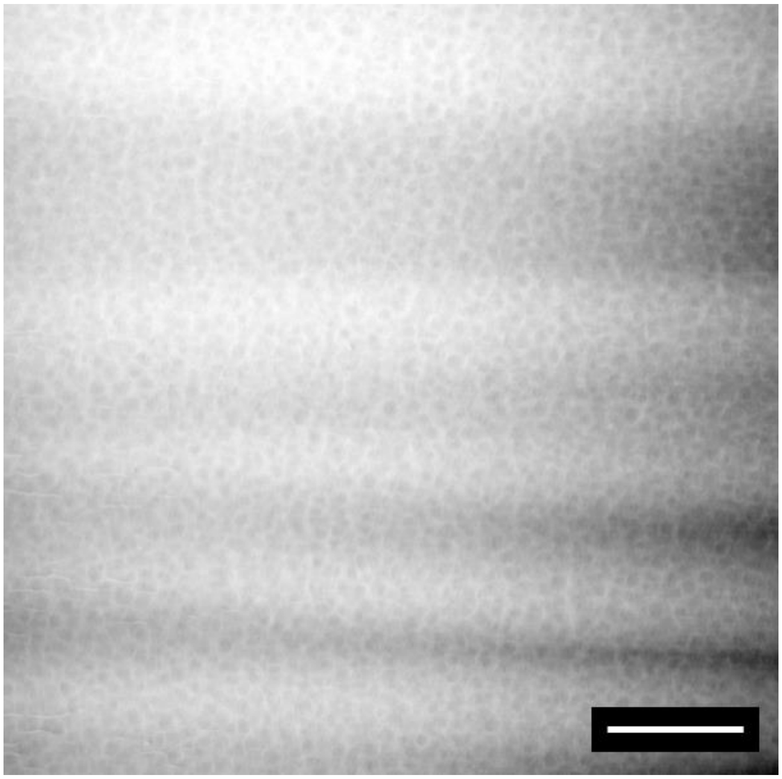

Supplementary Figure 5: Solutionized ME501 microstructure. HAADF-STEM image of ME501 sample solutionized at 1210°C for 1 hour. Scale bar, 500nm.

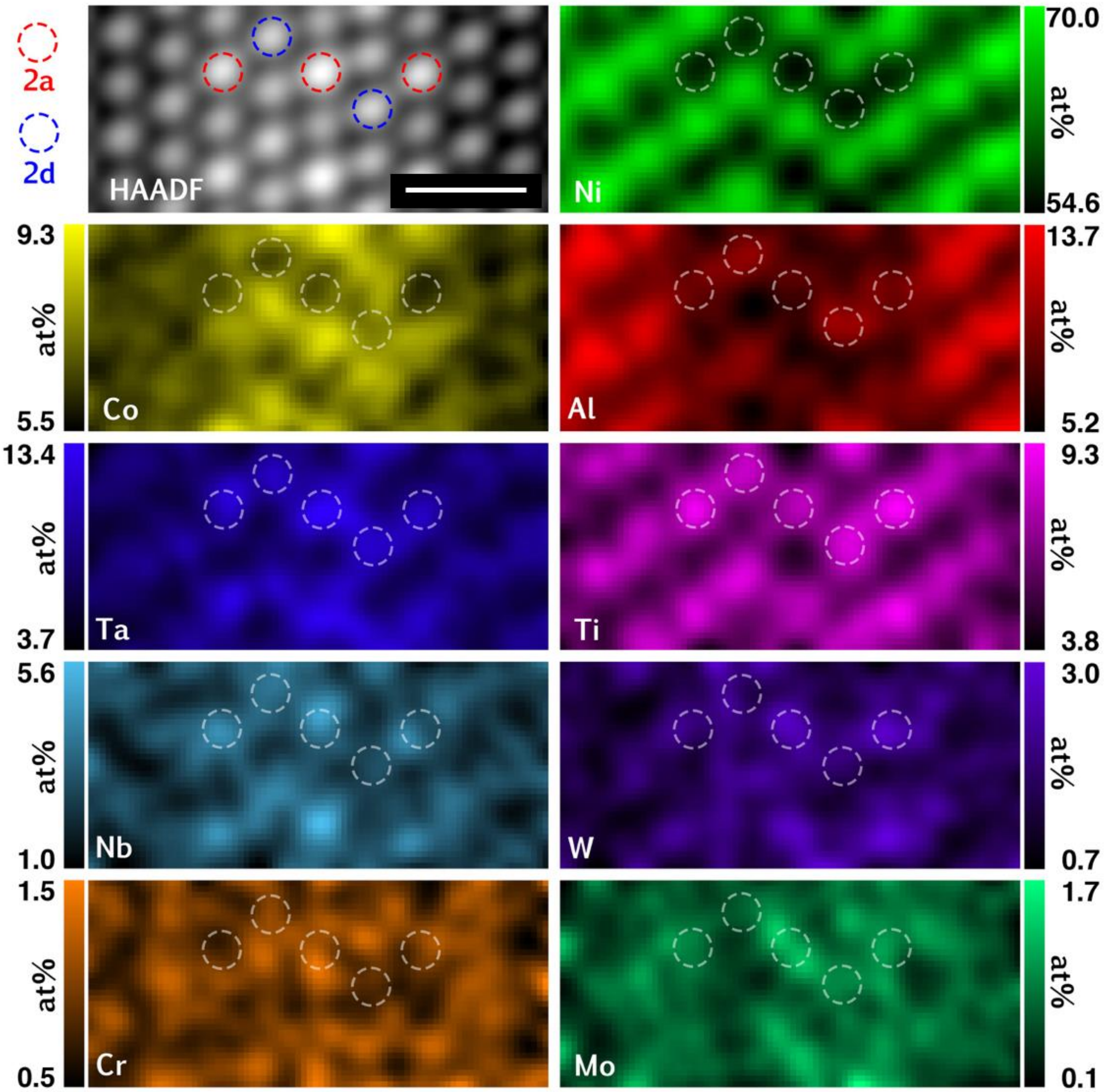

Supplementary Figure 6: Quantified EDX maps of SESF in ME501. Quantified atomic resolution EDX of η phase in γ′ showing the HAADF-STEM image of the fault exhibiting characteristic ordering of intensity within the fault; Ni sublattice (green); Co (yellow) segregating to Ni sites; Ta and Nb (dark and light blue, respectively) segregating to the Wyckoff 2a sites; Al and Ti (red and magenta, respectively) segregating to the Wyckoff 2d sites. W, Cr, and Mo (purple, orange, and light green, respectively) are fairly noisy. All EDX values are in at%. Scale bar, 0.5nm.

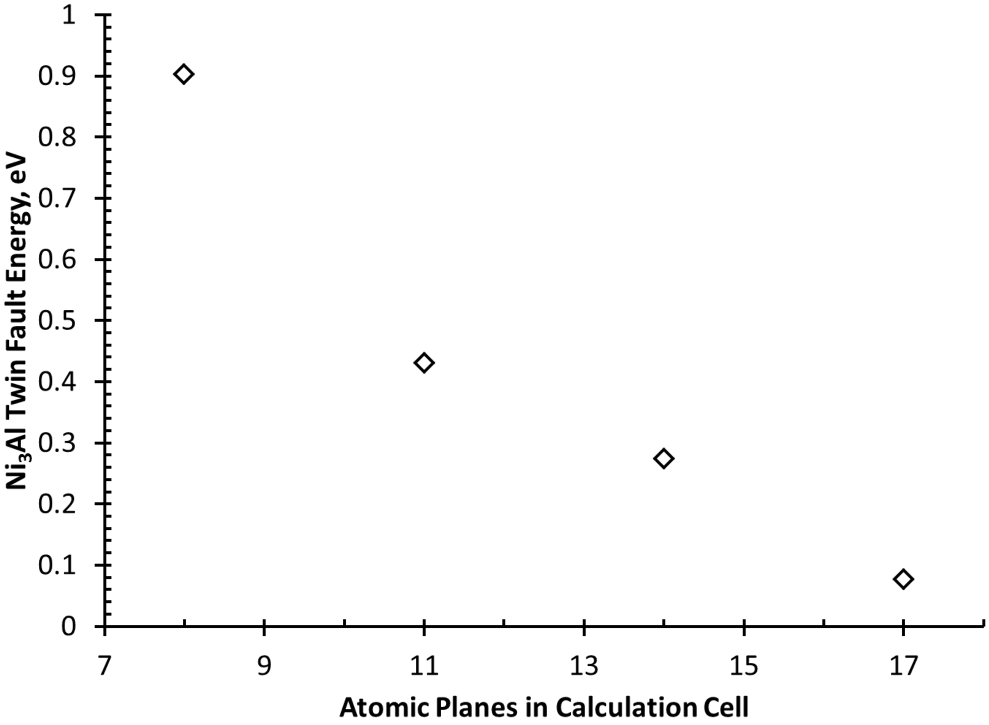

Supplementary Figure 7: Convergence study of twin formation energy. Twin energy in pure Ni_3Al calculated as a function of number of lattice planes in the calculation cell; the energetic cost of twinning decreases drastically as defects are separated by added lattice planes, as expected.

Supplementary Table 1: Differences in Alloy Chemistry. Alloying Differences in weight percent between ME3 and ME501

Alloy	Ni	Co	Cr	Mo	W	Nb	Ta	Al	Ti	Hf	C	B	Zr
ME3	Bal.	20.6	13.0	3.8	2.1	0.9	2.4	3.5	3.4	0	0.05	0.03	0.03
ME501	Bal.	18.0	12.0	2.9	3.0	1.5	4.8	3.0	3.0	0.4	0.05	0.03	0.05

Supplementary Table 2: Nanotwin formation frequency study. The twin occurrence rate for a 30μm^2 area for ME3 and ME501. Twins were found to be statistically more prevalent in ME3 than ME501.

Sample	Twin avg.	Twin std.	Twin std. err.	N
Un-deformed ME501	4.20	2.78	0.88	10
ME501	4.23	1.01	0.28	13
Un-deformed ME3	1.72	0.65	0.20	10
ME3	9.69	1.97	0.55	13

Supplementary Table 3: Nanotwin strain contributions. Amount of strain contributed to twinning for ME3 and ME501. Twinning played a significant role in ME3's creep performance.

Alloy	Strain from twinning	% of Total strain from twinning
ME3	0.0029	42%
ME501	N/A	0%

Supplementary Table 4: Determination of k-factors for EDX analysis. Experimentally determined k-factors for [110] ME501

Element	X-ray Series	⟨110⟩ k-factor	Error
Al	K	1.0334	± 0.01
Co	K	0.8694	±0.005
Cr	K	0.8372	±0.005
Mo	L	1.6450	±0.02
Nb	L	3.9240	±0.18
Ni	K	1.0000	--
Ta	M	5.8734	±0.15
Ti	K	0.8507	±0.01
W	M	2.4190	±0.05

Supplementary Table 5: Experimental error ranges for the atomic resolution EDX in supplementary figure 6.

**Element**	**Minimum value** ***(at%)***	**Maximum value** ***(at%)***
Al	1.0 ± 1.0	21.1 ± 5.1
Co	2.2 ± 1.0	15.3 ± 2.8
Cr	0.3 ± 0.4	4.8 ± 1.6
Mo	0.4 ± 0.4	5.8 ± 2.1
Nb	0.9 ± 1.0	12.6 ± 5.5
Ni	45.6 ± 4.7	81.6 ± 7.8
Ta	0.8 ± 0.8	21.0 ± 6.8
Ti	0.5 ± 0.6	15.6 ± 3.0
W	0.3 ± 0.3	7.5 ± 2.4

Supplementary Note 1. Chemistry and microstructural differences between ME3 and ME501

In **Supplementary Figure 1** are backscatter SEM images of the corresponding microstructures for ME3 and ME501. Even though both alloys experienced the same heat treatment, differences in alloying between the two resulted in different microstructures. ME501 has a finer microstructure, however, a higher γ' volume fraction. This higher volume fraction of secondary precipitates is a result of the greater amount of γ' formers (Ta, Ti, Hf, and Nb).[1] These elements are also known η phase formers.[2–5] **Supplementary Table 1** shows the composition differences between ME501 and ME3. The larger content of η formers in ME501 (13% of total weight percent) may explain why η phase nucleates in ME501 along SESFs while γ formers do so in to ME3 (9% of total weight percent).

7

Supplementary Note 2. Analysis of twin formation frequency during creep

Initial analysis using BF-STEM found nanotwins to be more active in ME3 compared to ME501, but the sample size reasonably obtained through STEM diffraction analysis of focus ion beam foils is not enough to conclusively make this assumption. Therefore, a technique called electron channeling contrast imaging (ECCI) was employed on both post creep and un-deformed samples to create a larger deformation mode sample size.[6,7] By using the backscattered electron detector at low magnifications, electron channeling pattern lines can be observed. These lines indicate diffraction conditions, that when activated through tilting the bulk sample, can allow diffraction contrast imaging of dislocations and/or faults. The ability to image defects at low magnifications from large bulk samples allows for a better statistical analysis. This technique was implemented on the [001] ME3 and ME501 samples to better understand the regularity of nanotwins in both alloys both before and after a creep test. Revealed in **Supplementary Figure 2** is an example of two ECCI images showing defects in ME3 and ME501 from post-creep samples. Faults that extend through both γ and γ' phases were classified as twins; whereas, those that were isolated to a precipitate were deemed stacking faults. **Supplementary Table 2** shows the final statistics from the ECCI investigation. As was discovered in the STEM diffraction work, nanotwins were much more prevalent in ME3 than in ME501. Two sample, student t tests were employed to investigate the null hypothesis that no twins were formed during the creep test (H_0: μ_1-μ_2=0) where μ_1 is the mean twin occurrence found in un-deformed samples and μ_2 is the mean twin occurrence observed in post-creep samples. A 0.05 statistical threshold was chosen for this study. Rejecting the null hypothesis would indicate that twins were either created or destroyed during the creep test, while not rejecting would reveal that no significant number of twins were formed during the test. The p value for H_A: μ_1-$\mu_2 \neq 0$ between the un-deformed and crept ME501 samples

was found to be 0.97 which signifies the null hypothesis is not rejected and no significant number of twins were formed in ME501 during the creep test. However, for ME3 the p value for H_A: $\mu_1-\mu_2\neq0$ was less than 0.0001 indicating that nanotwins had in fact been formed during the creep test.

Viswanathan *et al.*[8] revealed a method to calculate the relative strain contribution from twinning using the equation below.

$$\gamma = \frac{N_{twin}\Delta_{avg}}{L} \qquad \textbf{(1)}$$

Using the ECCI images, for example those in **Supplementary Figure 2**, a line can be drawn across the image with a length (L) that will cross a number of twins (N_{twin}). Using the average width of twins (Δ_{avg}) determined from high-resolution HAADF-STEM images of edge-on twins, the strain contribution from twins (γ) was calculated in ME3 as shown **in Supplementary Table 3**. Those values take into account the percentage of twins that were present before the creep test began. Twinning was found to contribute around 42% of the overall strain in ME3 while none of the strain in ME501 could be attributed to twinning. Therefore, the increase in creep strength found in ME501 can be directly related to inhibition of creep by twinning. However, not all of the creep property differences between the two alloys can be explained solely through this phase transformation strengthening mechanism. The higher amount of γ' formers in ME501 led to a larger volume fraction of secondary precipitates (52% compared to 47% in ME3), and the finer microstructure may have also contributed to the creep strength differences.. It is emphasized that this comparison of twinning propensity is based on comparison of behavior at significantly different stress levels for the two alloys, in order to achieve similar creep rates. In this way, we

have attempted to compensate for the volume fraction differences. Furthermore, while comparing coarse and fine microstructures in ME3, Unocic *et al.*[9] found that the finer microstructure possessed better high temperature properties compared to the coarser microstructure. They attributed the improvement of properties to the smaller channel widths found in the fine microstructure, which inhibited dislocation motion. However, these tests were conducted on polycrystalline samples and the relationship between microstructure and creep properties is still not clear. Diologent and Caron[10] found that increasing precipitate size, while keeping the volume fraction constant, improved the primary creep properties for single crystal AM1 and MC544. This improvement was attributed to a decrease of precipitate shearing dislocations in the larger precipitate samples. In this study, single crystal samples were tested, removing the complicating and confounding variables of grain boundaries, secondary phases along grain boundaries (ie carbides and borides), and grain size in order to more effectively explore the effects small alloying differences have on creep properties.

Supplementary Note 3. Quantification of EDX maps

As mentioned in the Methods section, 256 x 256 pixel atomic resolution EDX maps were collected in a region including an SESF in the ME501 alloy. Given the fact that the SESF forms the η phase, it can be asserted that site-specific segregation should exhibit the same periodicity dictated by the η phase crystal structure.[2,4] Following on this, repeating units along the SESF can then be extracted from the total spectrum image and summed to improve the signal-to-noise ratio. Because the SESF was not oriented parallel to any side of the original spectrum image, the entire spectrum image was rotated using a bicubic interpolation from the Matlab 2015a Image Processing Toolbox.[11] As seen in **Supplementary Figure 3**, 11 repeating units, corresponding to two unit cells in the $[11\bar{2}0]$ projection were extracted from the total spectrum image and then summed. Given the limited amount of SESF length available, six sequential repeat units were taken (yellow boxes), with five additional repeat units taken midway between each of the previous six. This results in overlapping data sets; however, this is still a valid process because of the symmetry of the region contained. If, for example, this assumption were incorrect, and the site-specific segregation were not occurring, the resulting summed data would exhibit a more uniform composition. By summing the 11 regions mentioned above, the EDX spectra at each pixel were vastly improved**. Supplementary Figure 4** shows a representative spectrum from the raw data (top) and summed data (bottom). The raw data displays very few counts per peak, which is typical of atomic resolution EDX data. While the summed spectrum image shows nearly an order of magnitude higher maximum peak counts and an even greater increase in total integrated peak counts. The summed spectrum image, with a better signal-to-noise ratio than the raw data, was then imported into Esprit and quantified using experimentally determined Cliff-

Lorimer k-factors. K-factors were experimentally found by solutionizing the ME501 alloy at 1210°C for 1 hour, seen in **Supplementary Figure 5**. Some variation in HAADF-STEM image intensity suggests that there may be a compositional modulation that was not fully solutionized; however, the EDX spectrum was taken from a large area on a ⟨110⟩ zone axis just like the atomic resolution EDX data to ensure that the proper channeling condition was taken into account. Experimental k-factors (wt%) for this alloy and the associated error with each can be seen in **Supplementary Table 4**. After the summed spectrum image was quantified using the Bruker Esprit software package, along with the experimentally determined k-factors, the final atomic resolution EDX maps were constructed, as seen in **Supplementary Figure 6**. Error in the EDX quantification is shown in at% in **Supplementary Table 5**. Values displayed in **Supplementary Table 5** are the minimum and maximum values (in at%) and their associated standard deviations.

Supplementary Note 4. DFT calculation limitations

Due to limitations in computational resources, a full convergence study of twin formation energy in ME3 and ME501 with respect to calculation cell size was not possible. In order to establish that the relatively high twin formation energies were a result of cell size limitations, we conducted a partial convergence study on twin formation energy on the simpler pure Ni_3Al system. Results are displayed in **Supplementary Figure 7** and indicate that no fewer than 20 {111} planes would be necessary to obtain converged formation energies consistent with twinning being a low energy defect in Ni_3Al.

Supplementary References

1. Jena, A.K., Chaturvedi, M. C. The role of Alloying Elements in the Design of Nickel-base superalloys. *J. Mater. Sci.* **19,** 3121–3139 (1984).
2. Asgari, S., Sharghi-moshtaghin, R. & Sadeghahmadi, Mehdi Pirouz, P. On phase transformations in a Ni-based superalloy. *Philos. Mag.* **93,** (2013).
3. Bouse, G. K. Eta and Platelet Phases in Investment Cast Superalloys. *Superalloys 1996 (Eighth Int. Symp.)* (TMS,1996).
4. Pickering, E. J. *et al.* Grain-boundary precipitation in Allvac 718Plus. *Acta Mater.* **60,** 2757–2769 (2012).
5. Long, F. *et al.* Formation of η and σ phase in three polycrystalline superalloys and their impact on tensile properties. *Mater. Sci. Eng. A* **527,** 361–369 (2009).
6. Coates, D. G. Kikuchi-like Reflection Patterns Obtained with the Scanning Electron Microscope. *Philos. Mag.* **16,** (1967).
7. Ahmed, J., Wilkinson, A. J. & Roberts, S. G. Characterizing dislocation structures in bulk fatigued copper single crystals using electron channelling contrast imaging (ECCI). *Philos. Mag. Lett.* **76,** 237–245 (1997).
8. Viswanathan, G. B., Karthikeyan, S., Sarosi, P. M., Unocic, R. R. & Mills, M. J. Microtwinning During Intermediate Temperature Creep of Polycrystalline Ni-Base Superalloys: Mechanisms and Modeling. *Philos. Mag.* **86,** 4823–4840 (2006).
9. Unocic, R. R. *et al.* Deformation Mechanisms in Ni-Base Disk Superalloys at Higher Temperatures. *Superalloys 2008 (Eleventh Int. Symp.)* 377–385 (TMS, 2008).
10. Diologent, F. & Caron, P. On the creep behavior at 1033K of new generation single-crystal superalloys. *Mater. Sci. Eng. A* **385,** 245–257 (2004).
11. Matlab and Imaging Processing Toolbox Release 2015a (The Mathworks, Inc., 2015).
